# SnO_2_ Highly Sensitive CO Gas Sensor Based on Quasi-Molecular-Imprinting Mechanism Design

**DOI:** 10.3390/s150203789

**Published:** 2015-02-05

**Authors:** Chenjia Li, Meng Lv, Jialin Zuo, Xintang Huang

**Affiliations:** Institute of Nanoscience and Nanotechnology, Department of Physical Science and Technology, Central China Normal University, Wuhan 430079, China; E-Mails: cjli@mails.ccnu.edu.cn (C.L.); lvmeng@mails.ccnu.edu.cn (M.L.); bryanzuo@mails.ccnu.edu.cn (J.Z.)

**Keywords:** SnO_2_ nanomaterial, molecular imprinting mechanism, CO gas sensor, high sensitivity

## Abstract

Response of highly sensitive SnO_2_ semiconductor carbon monoxide (CO) gas sensors based on target gas CO quasi-molecular-imprinting mechanism design is investigated with gas concentrations varied from 50 to 3000 ppm. SnO_2_ nanoparticles prepared via hydrothermal method and gas sensor film devices S_C_ (exposed to the target gas CO for 12 h after the suspension coating of SnO_2_ film to be fully dried, design of quasi-molecular-imprinting mechanism, the experiment group) and S_A_ (exposed to air after the suspension coating of SnO_2_ film to be fully dried, the comparison group) made from SnO_2_ nanoparticles are all characterized by XRD, SEM and BET surface area techniques, respectively. The gas response experimental results reveal that the sensor S_C_ demonstrates quicker response and higher sensitivity than the sensor S_A_ does. The results suggest that in addition to the transformation of gas sensor materials, surface area, and porous membrane devices, the Molecular Imprinting Theory is proved to be another way to promote the performance of gas sensors.

## 1. Introduction

As an *n*-type semiconductor, SnO_2_ has been extensively applied in gas sensing. SnO_2_ based nanomaterials have been widely investigated for the detection of various gases, including properties like fast-response speed, high chemical stability and prominent selectivity. A great deal of research has been dedicated to investigating the nanoparticles, nanotubes, nanowires, nanobelts and nanorods of SnO_2_ in the detection of carbon monoxide, hydrogen, methanol, nitrogen dioxide, and ethanol [1–10]. They attempt to improve the sensors' sensing performance by designing novel nano structures. Other CO gas sensors attempt to fabricate sensors with better performance by adopting methods like doping or using other metal oxide like Indium, since changing the containment of the material may distinctly improve the adsorption/desorption characteristics [11,12]. Accordingly, we choose to leave some marks on the material in advance; in other words, adopt the molecular imprinting method. Gas sensors are mainly applied to detecting toxic gases, flammable gas and medical diagnosis [13,14].

Its operating mechanism can be explained as follows. Oxygen ions absorbed in the surface of SnO_2_ can form a carrier depletion layer, which leads to a decrease in the electronic conductivity. Once exposed to reducing gas, the active substrate with oxygen adsorbates will react with the gas and enable the electrons injected back into the active material, causing an increase in electronic conductivity. During the procession, what determines the sensing performances is the diffusion of gas throughout the pores of the sensing layer [13].

For the purpose of improving the sensor performance, a large amount of research has focused on controlling SnO_2_ nanostructure, ignoring device fabrication procedures. As a matter of fact, the method of producing the sensing film can improve the porosity of the material, which will lead to better performance in the gas-sensing behaviors of sensors [15].

Hence, we introduce molecular imprinting technology [16]. Through this technology, the material can be prepared with high selectivity and specificity. Essentially, the gas sensing theory is the process of the absorption of gas on the metal oxide semiconductor surfaces and charge redistribution between the surfaces and the absorbed gas molecules. We here follow the mechanism in the fabrication of gas sensors. Template-shaped cavities are created by molecular imprinting on the surface of the material with a record of the size, structure, and other physicochemical properties of the template molecules to be used in molecular recognition. Molecular imprinting is a technique commonly used for the fabrication of biomimetic polymeric recognition sites or “plastic antibodies/receptors”, which has attracted extensive interest recently. This technique makes exaltation with higher efficiency and selectivity possible, leading to the development of some remarkable features, such as target identification and selectivity, which can be introduced into the design of ultra-sensitive gas sensors [17–21]. Molecular imprinting technique has been widely used in biosensing [22], producing specific separation materials [23] and making selective binding site for specific chemicals [24]. According to the above, we suggest that the similar mechanism, the quasi-molecule-imprinting method, can be used in fabrication of CO gas sensors. When CO comes into contact with the surface of the material, the CO cluster creates a “hole”, which records the “shape” of the CO cluster. Thereby, next time CO is in contact with the material, the sensors demonstrate better adsorption/desorption performance.

Thus, based on the molecular imprinting technique mechanism, we designed highly sensitive SnO_2_-based carbon monoxide gas sensors. CO molecules were introduced to the device fabrication process to acquire a certain structure which is more suitable for the adsorption and desorption of CO gas. We simply borrow this mechanism and conducted our research based on the simplest scenarios. We tried our best to exclude interference such as the thickness of the film, the temperature, the humidity, *etc.* (which is not our focus in the research). Furthermore, our target is to examine whether molecular imprinting can independently improve the sensing performance of CO gas sensors.

To make our narrative clearer, first, we use the hydrothermal method to prepare SnO_2_ mesoporous nanomaterial. Then, the prepared SnO_2_ film sensors were divided into two groups, which were named S_A_ and S_C_, respectively. Sensors in S_C_ were dried in a carbon monoxide (which is the target gas) atmosphere to imprint CO on the surface of the material, and sensors in S_A_ were dried in the air to make a comparison group. During the gas response test, two types of SnO_2_ sensors are exposed to CO gas with various concentrations ranging from 50 to 3000 ppm. The results show that the sensor S_C_ exhibits a quicker response/recovery speed and higher response compared with S_A_ in all CO concentrations. In addition, a quicker response/recovery speed of S_C_ compared with S_A_ is more obvious at low CO concentration, while a higher sensitivity of S_C_ compared with S_A_ is more evident at high CO concentration. It demonstrates that adopting the molecular imprinting technique in the gas film device fabrication process can enhance gas sensor performance remarkably, in addition to gas sensor materials, surface area and porous structures of the device film.

## 2. Experimental Section

### 2.1. Synthesis of SnO_2_ Nanomaterials

All the reagents in this work were analytically pure and used without any further purification. SnO_2_ mesoporous nanomaterials were synthesized by hydrothermal method [13].

In a typical synthesis, 1 g of tin (II) chloride (SnCl_2_·2H_2_O) was dissolved in 70 mL of distilled water with continuous magnetic stirring in a glass beaker (100 mL). Then, 0.5 mL of hydrochloric acid (38%) was dropped into the white suspension above with sufficient stirring at room temperature about 2 h until the solution became clear.

After that, the mixture solution was transferred into Teflon-lined stainless steel autoclaves, which was later sealed tightly, heated and maintained at 90 °C for 20 h. After the hydrothermal reaction, the mixture turned yellow and the mixture was centrifugated to attain the yellow precipitates. The yellow precipitate should be washed with distilled water for several times, and finally dried in air at 60 °C. The yellow precursors were calcinated at 350 °C in air for 2 h. Finally, the light yellow mesoporous SnO_2_ nanoparticles were obtained.

### 2.2. Device Fabrication

Gas sensors were fabricated as follows: The as-prepared powder was mixed with water to form a paste. Then the paste was coated onto a ceramic tube (axial length 4 mm and basal diameter 1.5 mm) to form a thick film. The thickness of the sensing film was about 100 μm. The ceramic tube was equipped with two pairs of Au electrodes and a Ni–Cr heater through the tube to control the experimental temperature. Then we divided the sensor into two groups. One was dried in CO gas condition (sensor sample S_C_) at room temperature for 12 h. The other was dried in air (sensor sample S_A_) to make a comparison group. Table 1 lists the average pore size and the Brunauer-Emmett-Teller (BET) surface area of two samples, respectively.

### 2.3. Characterization

**Figure 1.**
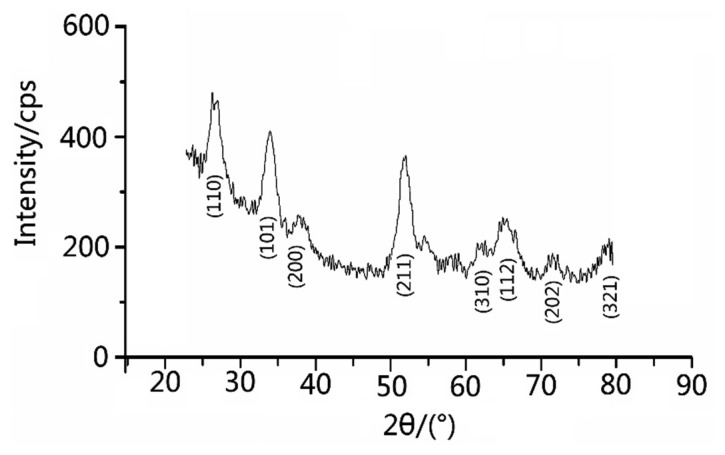
XRD pattern of as-prepared SnO_2_ nanomaterial.

The products were characterized by scanning electron microscopy (SEM, JSM-6700F, JEOL Ltd., Akishima Musashino, Japan; 5 kV). X-ray power diffraction (XRD, Brucker D-8 Avance, BRUKER AXS GmbH, Karlsruhe, Germany, Cu Ka, λ = 1.5418 Å) analysis was conducted to characterize the products in the range of 20°–80°. The porous structure had been further confirmed by the Nitrogen adsorption–desorption analysis (Belsorp Mini, Ankersmid Ltd., Nijverdal, Netherlands). Figure 1 shows the X-ray diffraction (XRD) experiment map of the material. In the XRD pattern, we could barely see any characteristic peaks of any impurities. The fluctuation were possibly caused by noise in the instruments or minor impurities which could be ignored. Such a pattern shows that the SnCl_2_ have deformed to SnO_2_ nanoparticles.

**Figure 2.**
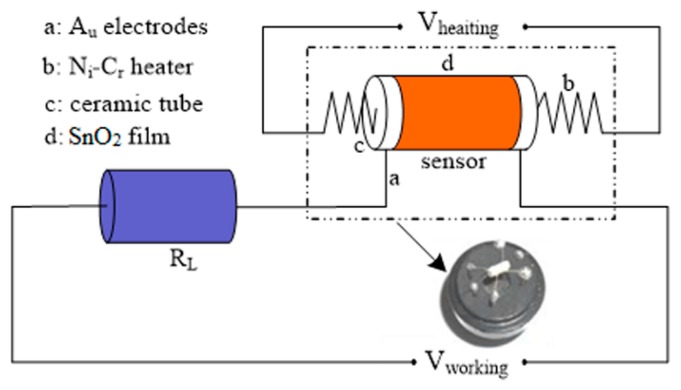
Circuit diagram of the gas sensing test system.

Figure 2 shows the schematic diagram of the gas sensing instrument. A static process was used to test the properties of sensors in a 10 L chamber. The test atmosphere was prepared by injecting a given amount of saturated CO gas into the 10 L chamber, where an air pump was fixed to homogenize the CO gas in the chamber. Sensors were inserted onto a detecting stick which could be expediently inserted into the test chamber for the measurement of sensing performance. The gas sensing properties were detected by a computer-controlled Navigation 4000-NMDOG gas-sensing measurement system (Zhongke Micro-Nano Technology Co., Ltd., Beijing, China), and the resistance of the gas sensor in clean air (R_a_) together with target gas (R_g_) could be monitored in real time respectively. The gas response of the sensor was defined as R_a_/R_g_ for reducing gas. After each test, the chamber was flushed with clean air for 20 min to reset the testing atmosphere.

## 3. Result and Discussion

### 3.1. Structure Analysis

**Figure 3.**
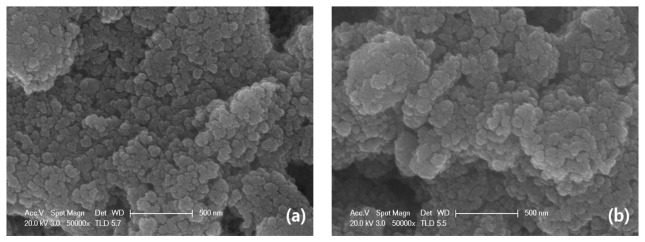
SEM images of (**a**) S_A_; (**b**) S_C_.

First, we examined the purity of SnO_2_ nanomaterial. Figure 2 shows the XRD pattern of as-prepared SnO2 nanomaterial. According to the JCPDS file No. 41-1445, the distinguishable characteristic peaks on the map well corresponded to the characteristic peaks of SnO_2_ in the file and there were no characteristic peaks of any other impurities. The broad and low diffraction peaks exhibited the small crystallite size of as-prepared samples and films [25]. Figure 3 reveals the uniform particle size of SnO_2_ nanomaterial films that were dried in air and CO, respectively. In fact, the pictures taken by scanning electron microscope (SEM) do not show obvious differences between the samples (S_A_ and S_C_). While this is exactly the special feature of the molecular imprinting mechanism, which means that the differences in the micro-structures of S_A_ and S_C_ may actually lie within a molecular or atomic scale.

**Figure 4.**
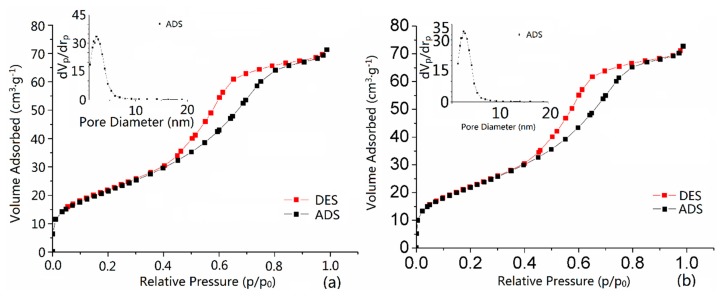
Nitrogen gas adsorption-desorption isotherms and BJH pore size distribution (inset) of (**a**) S_A_; (**b**) S_C_.

Then, the adsorption-desorption isotherm research is casted in order to have a better understanding of the properties of pores and calculate specific surface area and pore size distribution. According to the IUPAC recommendations [26], the experimental gas adsorption-desorption isotherms are normally categorized into six categories. Additionally, the adsorption-desorption hysteresis loop is divided into four types. In our research, N_2_ adsorption-desorption analysis confirmed distinctively the porosities of nanoparticles. In Figure 4a,b, SnO_2_ nanomaterial film device S_A_ and Sc have the adsorption-desorption isotherm shape of IV with the hysteresis loop type of H1. It is usually attributed to the thermodynamic or/and network effects. The type H1 is often reported for materials that consisted of compacts of approximately spherical particles arranged in a fairly uniform way [26,27]. Additionally, the H1 hysteresis loop accounts for materials with cylindrical pore geometry and a high degree of pore size uniformity and indicates that the facile pores are in a 3D intersection network [28]. Moreover, Table 1 shows that the average pore sizes are 5.5383 and 5.5696 nm for sample S_A_ and S_C_, respectively. The BET (Barrett-Joyner-Halenda) surface area of S_C_ (79.636 m^2^ g^−1^) is slightly larger than that of S_A_ (80.786 m^2^ g^−1^). All the differences above, the hysteresis loops, the pose size distributions and surface areas of S_A_ and S_C_, indicate that CO had indeed affected the film device nanostructures. By consequence, these results may enhance the performance of sensors dried in the target gas CO. We call it a SnO_2_ highly sensitive CO gas sensor based on a molecular imprinting mechanism design. To our knowledge, the design concept of our semiconductor resistant CO gas sensor is reported for the first time.

### 3.2. Gas-Sensing Properties

In our research, we consider sensitivity and response/recovery time as the two most important factors of sensors' responding performance.

**Figure 5.**
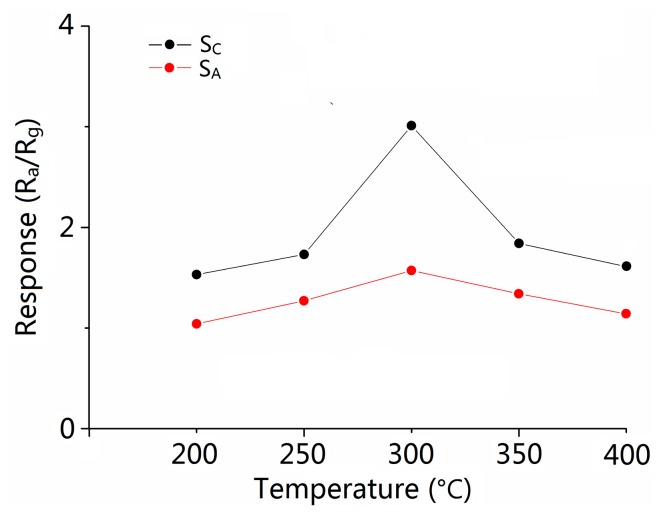
Response (R_a_/R_g_) for different samples to 500 ppm CO gas at various operating temperatures.

The operating temperature is an important parameter for the semiconductor oxide sensors [29,30]. In order to determine the optimum operating temperature, the temperature-dependent response measurements with 500 ppm CO gas of different sensors were performed at temperatures ranging from 200 °C to 400 °C. Obviously in Figure 5, at 300 °C, the gas sensors show the best response performance. Thus, 300 °C was chosen as the optimal operating temperature for SnO_2_ based CO gas sensing studies.

**Figure 6.**
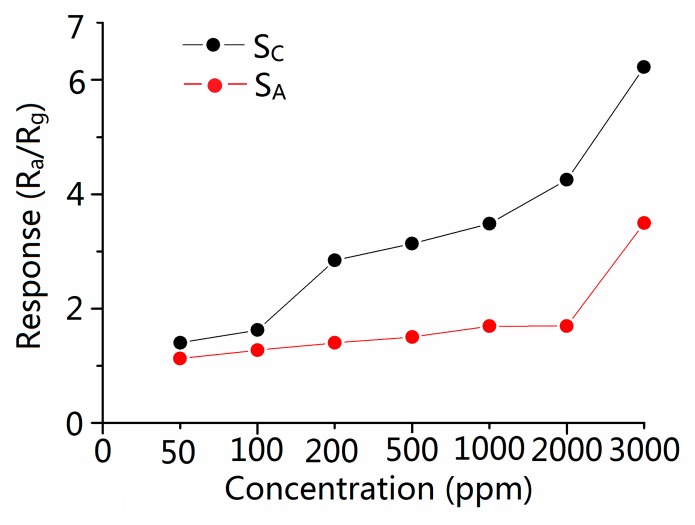
Response of sensor S_A_ and S_C_ at 300 °C *versus* CO concentrations.

**Figure 7.**
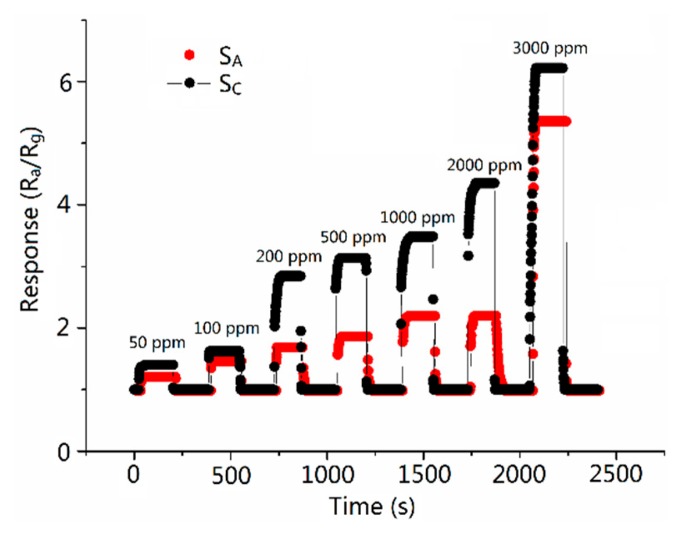
Responses to 50, 100, 200, 500, 1000, 2000, 3000 ppm of CO gas for S_A_ and S_C_ at 300 °C.

Figures 6 and 7 show the sensitivity of sensors *versus* the CO concentration ranging from 50 to 3000 ppm at the operating temperature of 300 °C. It is obvious that the sensitivity increases when the concentration increases and the response of S_C_ is higher than S_A_ in the all test ranges of CO concentration. The response of S_C_ toward 500 ppm CO reached the value of 3.13, which is about 2.1 times higher than the response of S_A_. Table 2 shows the response time of 2.6–12.2 and 3.012–15.875 s (the response/recovery time is defined as the time the sensors take to complete 90% of total signal change), and recovery time of 3.6–14.8 and 3.7–16.4 s at various CO gas concentrations for sensors S_C_ and S_A_, respectively. It is obvious that S_C_ has a shorter response/recovery time compared with S_A_ at various CO gas concentrations. The shortened response/recovery time and the promoted sensitivity show that because of the design of the molecule imprinting mechanism, the response performance of the sensors has been remarkably improved. Moreover, our research focuses on inventing a new path (borrowing the mechanism of the molecular imprinting method) in improving semiconductor sensors' sensing performance. Therefore, the mechanism can be applied to various kinds of nanomaterial. No matter which method the CO gas semiconductor sensor has previously used, adopting our method can further promote the sensing ability of the sensors.

As we can see in Figures 6 and 7, at comparatively lower concentrations (lower than 500 ppm), the promotion of S_C_'s response is subtle while the response time and the recovery time change to a considerable extent, while on the map, we can barely see the changes in the response time and the recovery time between S_C_ and S_A_. We here make a presumption that at lower concentrations, compared with that at higher concentrations, CO is so thin that it acted on and left S_A_ slowly. While because of the imprinting method mechanism, S_C_ responds to and recovers with CO immediately. Therefore, we can observe the change in response and recovery times. It fully testifies the improvement in recovery and response times in S_C_. While at higher concentrations, the CO acted on and left the sensors quickly in the cases of both S_C_ and S_A_. The disparity between the times still exists but can barely be observed. However, because of the elevation in CO concentration, the manifestation of S_C_'s response is magnified. A more palpable improvement in response performance of Sc can be observed than that at lower concentrations.

**Figure 8.**
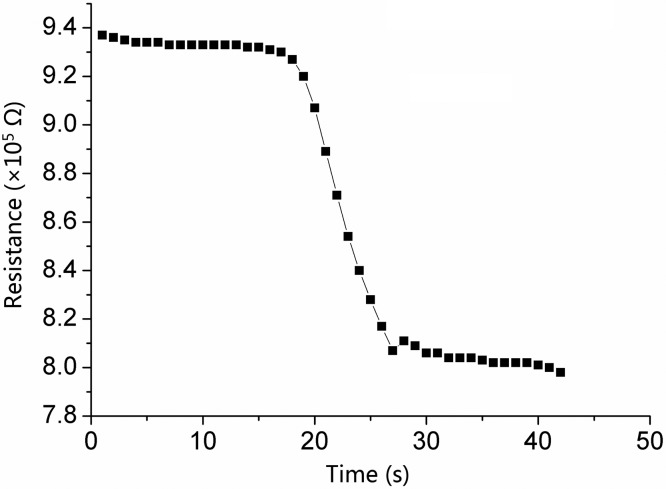
Response transients to 5 ppm CO gas for S_C_ at 300 °C (the detection limit of S_C_).

We also examined the detection limits of the sensors. Detection limit is the minimal detecting gas concentration that acted on the sensors. It is another vital factor determining semiconductor sensor's performance [31,32]. In Figure 8, there is a distinct response at a CO concentration of 5 ppm, while at lower concentrations, we can barely see the resistance change. Therefore, we assert that the detection limit of gas concentration is 5 ∼ ppm.

**Figure 9.**
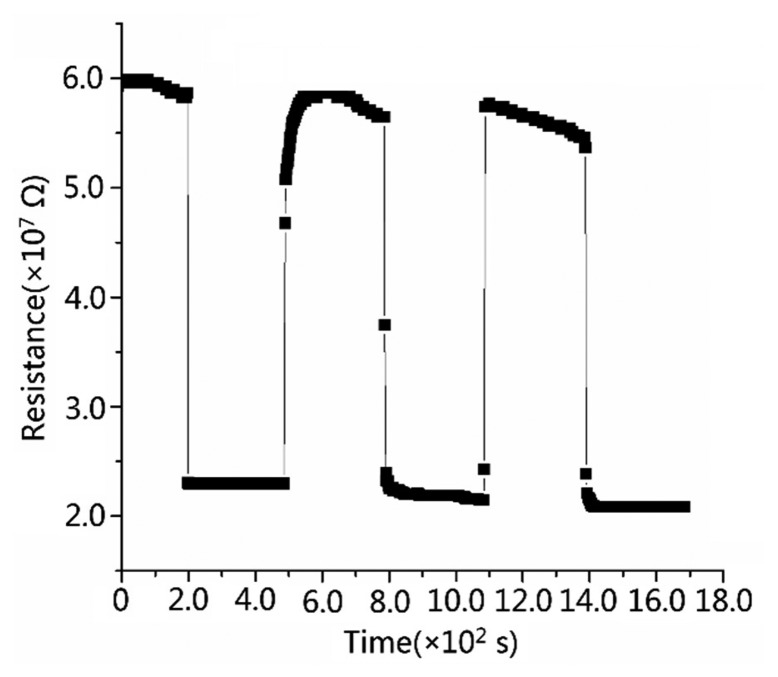
Response transients to 1000 ppm CO gas for S_C_ at 300 °C

Additionally, the “baseline drift” phenomenon can also be observed in Figure 9. It generally shows that the recovery resistance after each measurement cannot return to that of the last measurement. Moreover, the platform of R_a_ decreases in recovery curves, which has been reported in previous research [13,15,33,34]. Specially, at lower concentrations of CO, the R_a_ does not decrease greatly in the recovery period. Additionally, the unobvious “baseline drift” phenomenon at low concentrations fully testifies that Sc demonstrates an excellent adsorption and desorption performance in response to CO.

**Figure 10.**
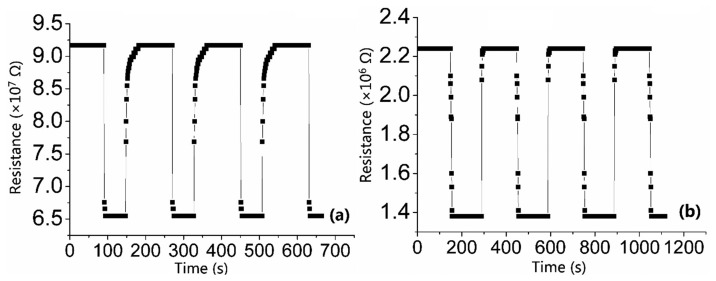
Repetitive response and recovery of (**a**) S_A_; (**b**) S_C_ to 50 ppm CO gas at 300 °C.

Stability and repeatability should also be examined for gas sensors in terms of practical applications. In Figure 10, the sensors testing atmosphere cycles between clean air and 50 ppm CO. The stable and repeatable characteristics can be reflected in the reversible cycles of response and recovery curves. Besides, as is mentioned in previous sections, S_C_ has a shorter recovery time than S_A_. Figures 9 and 10 also indicate that “baseline drift” has nothing to do with the repeated tests.

### 3.3. Sensing Mechanism

According to the basic gas-sensing theory, the gas molecules adsorbed on metal oxide semiconductor surfaces then changes on nanomaterial surfaces and the molecules redistribute. Specifically, the material's surface adsorb the oxygen molecule and form the oxygen adsorbates such as O^2−^, O_2_^−^ and O^−^. The electrons are from the *n*-type active material of the oxygen adsorbates. By consequence, the structural changes in such electron-depleted surface layers result in the reduction in the electronic conductivity of the metal oxide *i.e.*, SnO_2_ in this research. The reducing gas, such as CO, plays the role of the gas molecules mentioned above. The reduction reaction enables the active material to obtain electrons, which results in an increase of electronic conductivity of active material [3].

Thus, the performance of sensors is mainly determined by the adsorption and desorption of CO in this research. CO cluster interacts with the as-prepared material when the sensor is put into CO atmosphere for 12 h, which optimized the pore structure of SnO_2_ in order to exhibit better adsorption and desorption performance of CO. This process of imprinting nanomaterials (SnO_2_) by CO clusters is similar to the mechanism of the molecular imprinting method (defined as quasi molecular-cluster imprinting mechanism). Table 1 shows that the average pore size of the film CO is about 4.3 nm, which may be the critical size for the smooth adsorption and desorption of CO gas. However, further relevant research on the mechanism is still in progress.

## 4. Conclusions

In this research, we first follow the traditional synthesis process of SnO_2_ semiconductor gas sensors. Then, in the device fabrication process, we dry one group of sensors (S_C_) in CO, while drying another group (S_A_) in air. The results show that compared with S_A_, the sensor Sc exhibits a stronger response and shorter response/recovery time, suggesting that it is plausible to introduce the molecular imprinting mechanism to device fabrication. It also reveals that our method of combining the molecular imprinting mechanism with the hydrothermal method may be a new route to designing highly sensitive gas sensors.

## Figures and Tables

**Table 1.** Average pore size and Brunauer-Emmett-Teller (BET) surface area of S_A_ and S_C_.

**Sample**	**Average Pore Size (nm)**	**BET Surface Area (m^2^ g^−1^)**
S_A_	5.5383	79.636
S_C_	5.5696	80.786

**Table 2.** Response and recovery time of different samples under various CO gas concentrations.

**Gas Concentration (ppm)**	**Response Time (s)**		**Recovery Time (s)**	
	
	**S_C_**	**S_A_**	**S_C_**	**S_A_**
50	12.266	15.875	14.877	16.456
100	8.087	10.949	12.99	13.892
200	7.219	10.834	10.242	12.4
500	5.484	7.13	7.965	8.706
1000	4.45	5.773	5.186	6.499
2000	3.252	3.448	3.758	3.929
3000	2.659	3.012	3. 625	3. 701
